# Palliative care professionals’ perceptions of their skills and the implementability of evidence-based bereavement care guidance: A cross-sectional survey study

**DOI:** 10.1177/26323524251369119

**Published:** 2025-09-16

**Authors:** Sina Gyarmathy, Marco Riguzzi, Rahel Naef

**Affiliations:** 1 ZHAW Zurich University of Applied Sciences, School of Health Sciences, Winterthur, Switzerland; 2 Institute for Implementation Science in Health Care, Faculty of Medicine, University of Zurich, Switzerland; 3 Centre of Clinical Nursing Science, University Hospital Zurich, Dübendorf, Switzerland

**Keywords:** evidence-based practices, implementation, palliative care, bereavement, family, healthcare staff, death, hospital

## Abstract

**Objective:**

Bereavement support is recommended in specialised palliative care, but it is often underdeveloped. This study assessed palliative care professionals’ (PCPs’) skills in family care, their attitudes towards evidence-based practices, and their perceptions of a new, evidence-based bereavement care guidance before its implementation.

**Methods:**

A cross-sectional study was conducted between September and December 2023 in specialised palliative care services in two Swiss hospitals. Seven validated instruments were used to assess PCPs’ skills in family care (FNPS, EPCS, and ICS-Nurse), attitudes towards evidence-based practices (EBP-B), and perception of a newly developed, evidence-based bereavement care guidance (acceptability AIM, appropriateness IAM, feasibility FIM). The data were analysed using descriptive and nonparametric methods.

**Results:**

The 39 participants (response 63%; 28 nurses, 6 physicians, 5 others) rated their skills in family care as high (median ⩾75% of scale range) and had an open attitude towards evidence-based practices (median 64%). They perceived the bereavement care guidance as highly acceptable and appropriate (median 75%) and rather feasible (median 68%). A more favourable attitude towards evidence-based practices was associated with higher perceived acceptability (Spearman’s rho, *p* = 0.038), appropriateness (Spearman’s rho, *p* = 0.029), and feasibility (Spearman’s rho, *p* = 0.0019) of the guidance. Acceptability, appropriateness, and self-assessed skills (FNPS) depended on the local context (Mann-Whitney *U* test, *p* ⩽ 0.022).

**Conclusion:**

This study shows that PCPs rate their skills in working with families at the end of life as high and perceive evidence-based bereavement care as implementable. An open attitude towards evidence-based practices supports more favourable perceptions of new bereavement care guidance before its implementation.

## Introduction

### Background

Families of people who die experience distress during illness and dying^1^ that may persist after the patient’s death, including symptoms of depression, lack of happiness, increased anger, and decreased quality of life.^2^ While most can cope with the loss through support from family, friends, and community-based support structures, some develop adverse mental health outcomes, and one in ten bereaved people develop prolonged grief disorder, a mental health disorder recently included in diagnostic manuals.^3,4^

The World Health Organization^5^ advocates for emotional and social support during terminal illness and bereavement to mitigate adverse mental health outcomes, which is most frequently provided in hospices and specialised palliative care institutions^6^ to family members of cancer patients,^7^ most commonly by psychologists or nurses,^8^ with telephone counselling being the most common form.^6,8^

In specialised palliative care, a multidisciplinary team of specially trained healthcare professionals provides relief from the symptoms, pain, and distress of serious illness to patients.^9^ Family bereavement support interventions are defined as all services provided to family members during palliative care and after the death.^1^

Despite several bereavement care practice guidelines available for specialist palliative care services,^1,10^ bereavement care is not fully embedded as a routine service, and when it is provided, it often is not informed by current research and evidence-based guidance, as shown in an international overview by Lichtenthal et al.^11^ and specific studies from several Western countries.^7,12^ Overall, only around 50% of bereaved family members receive some form of bereavement support.^7,8^

Several studies have investigated the reasons for this lack of uptake and identified three domains: individual factors related to palliative care professionals (PCPs), institutional/systemic factors, and guideline-related factors.^13^ In particular, PCP-related barriers such as lack of training or skills or fear of being too close to those affected^12,14^ hinder implementation. Institutional/systemic barriers include a lack of time^12,14,15^ or organisational support.^12^ Guideline-related factors include uncertainty about who should be contacted^14^ or who should provide bereavement care.^12^

It is unknown, however, how such PCP-related barriers are associated with the PCPs’ perception of the implementation of evidence-based bereavement care, and what role their attitudes towards evidence-based practices (EBP) play.^13,16^ The existing knowledge and the lack of implementation suggest a “know-do gap.”

To address this know-do gap, we developed the BEST Care project that uses implementation science methods^16^ to assess PCPs’ perceptions of their skills and the implementation of an evidence-based bereavement care guidance (BCG), which was developed in collaboration with PCPs based on a contextual analysis^17^ and a survey of bereaved families.^18^ The BCG encompasses compassionate care and the active involvement of family members. Specific components are recorded in checklists by staff: providing specific information about the dying process and grief, conducting a risk assessment, sending a condolence card, making a follow-up phone call by nursing staff, and, if necessary, referring the family to community support services.

### Objectives

This study aimed to determine the extent to which PCPs (1) feel that they have the *skills* to care for bereaved families, (2) have a generally open *attitude* towards EBP that favours implementation, and (3) *perceive* an evidence-based BCG as acceptable, appropriate, and feasible. Furthermore, to ensure successful interprofessional collaboration in bereavement care, it aims to (4) identify and understand any *differences* between the involved professions and palliative care services.

## Methods

### Study design

This study was part of the multi-phase implementation research project “BEST for Family”. A quantitative exploratory cross-sectional survey design was used to answer the research questions following the “STROBE” statement.^19^ Following the Conceptual Model of Implementation Research,^20^ the PCPs were surveyed for the implementation outcomes of acceptability, appropriateness, and feasibility, and the service outcomes of quality of family care and skills of PCPs in family care pre-implementation, together with their baseline attitudes towards EBP.

### Setting and participants

The study was carried out in specialised inpatient palliative care services in two large hospitals in two major cities in the German-speaking part of Switzerland. Participants were PCPs (nurses, physicians, physiotherapists, occupational therapists, psychologists, social workers, chaplains, and nutritional therapists) who had direct contact with patients and their families in the final stages of life, dying, and/or during bereavement. PCPs were excluded if they had not yet completed their education, were not of full legal age, or had only worked in the palliative care service for less than 3 months. All eligible PCPs from the two services were invited to participate.

As this was an exploratory cross-sectional study, power calculations were not performed in advance.

### Data collection

The online questionnaire was distributed to potential participants via email by the contact persons (PCP or managers) of the respective services. The same information about the study and the same email and questionnaire were used in both services. Data was collected between 25 September and 31 December 2023. Two reminder emails were sent, and a video presentation and flyers were provided by the study team. In addition, personal reminders were given by the contact persons in the services.

### Study endpoints and measurement

A detailed description of each instrument, its scoring and psychometric properties is given in Table 1.

**Table 1.** Outcomes measures.

Domain	Measure	Details of measure	Background, validation, and references
Skills	FNPS^21^	Assessing the perception of confidence, skills, and knowledge in family care, the FNPS has 10 items with a 5-point Likert scale (1 = “high” to 5 = “low”; 1 = “agree” to 5 = “disagree”; 1 = “always” to 5 = “never”) and three open-ended questions (not included in this study). The scale is reverse coded: a lower mean score means high skills, confidence, and knowledge.	Translated and validated in German version Cronbach’s alpha: 0.84 ^26^
	EPCS^22^	Investigating the training needs of PCP in palliative and end-of-life care, the EPCS consists of 12 items answered on a 5-point Likert scale (0 = “not at all” to 4 = “very much.” A higher mean score indicates lower training needs.	Cronbach’s alpha in English: 0.96.^27^ Used in German^28^
	ICS-Nurse^23^	Investigating individualised care, the ICS-Nurse consists of 17 questions with a 5-point Likert scale (1 = “strongly disagree” to 5 = “strongly agree”), with a sum score ranging from 5 to 85. A higher total score reflects more individualised care.	Cronbach’s alpha in English: 0.88 ^23^
Attitude	EBP-B^24^	Quantifying the attitude towards evidence-based practice, the EBP-B questionnaire has 16 items answered on a 5-point Likert scale (1 = “strongly disagree” to 5 = “strongly agree”), with a sum score ranging from 16 to 80. A higher sum score indicates a more open attitude and stronger beliefs towards evidence-based practice. Reverse coding is used for two questions.	Translated and validated in German Cronbach’s alpha: 0.85 ^29^
Perceptions	AIM	To measure acceptability, appropriateness, and feasibility, three equivalently structured questionnaires were used. Each questionnaire contained four items recorded on a 5-point Likert scale (1 = completely disagree to 5 = “completely agree),” with a sum score ranging from 4 to 20. A higher sum score reflects higher acceptability, appropriateness, or feasibility of the intervention.	Translated and validated into German Cronbach’s alpha: 0.91–0.97 ^30^
	IAM		
	FIM^25^		

AIM: Acceptability of Intervention Measure; EBP-B: Evidence-Based Practice Belief Scale; EPCS: End-of-life Professional Caregiver Scale; FIM: Feasibility of Intervention Measure; FNPS: Family Nursing Practice Scale; IAM: Intervention Appropriateness Measure; ICS-Nurse: Individualised Care Scale for Nurses; PCP: Palliative Care Professional.

The key *skills* required for PCPs to provide bereavement care were assessed as follows. The “Family Nursing Practice Scale (FNPS)”^21^ measured the PCPs’ skills in working with families as a unit/system. The “End-of-Life Professional Caregiver Scale (EPCS)”^22^ assessed the PCPs’ palliative care knowledge and skills in supporting families in the specialised palliative care setting. The “Individualised Care Scale for Nurses (ICS-Nurse)”^23^ assessed the PCPs’ ability to tailor care to meet the needs and preferences of an individual and engage them.

The PCPs’ *attitudes* towards EBP were surveyed using the “Evidence-Based Practice Belief Scale (EBP-B).”^24^

To understand the PCPs’ *perceptions* of BCG implementation, it is recommended to assess implementation outcomes that can be evaluated before the actual implementation begins.^20^ In this study, the antecedent implementation outcomes of acceptability, appropriateness, and feasibility were selected to represent the providers’ opinions, using the “Acceptability of Intervention Measure (AIM),” “Intervention Appropriateness Measure (IAM),” and “Feasibility of Intervention Measure (FIM)” questionnaires.^25^

These questionnaires have different foci and complement each other, ensuring a comprehensive assessment of family caregiving skills, attitudes towards EBP in general, and perceptions of the implementability of specific BCG.

### Statistical methods

The data were analysed using R Statistical Software (v 4.1.2). Any inconsistent responses (e.g. regarding work experience or continuing education) were discarded. Only complete datasets were included in the analysis. Sociodemographic data were analysed descriptively. Spearman’s correlation coefficient was calculated to analyse the pairwise relationships between self-assessed skill measures, attitudes towards EBP, and perceptions of implementability (statistical hypotheses in Supplemental Material 1). Due to the small group sizes, three professional groups were created for further analysis: nurses, physicians, and other healthcare professionals. Pairwise comparisons were performed using Dunn’s Test with Bonferroni adjustment to assess group differences in ordinal data between professional groups. The Mann-Whitney *U* test was used to assess group differences between nurses with or without further education (continuing education programme in palliative care, CAS or MAS) and between palliative care services (A vs B). The significance level was set at α = 0.05 (critical type one error rate).

### Ethics

The responsible Ethics Committee of the Canton of Zurich waived the requirement for approval (BASEC number: Req-2023-00439). The study was conducted in accordance with good clinical practice guidelines.^31^ Participants gave informed consent before answering the questionnaire.

## Results

### Participants

A total of 62 PCPs from both services were invited to participate, of whom 52 PCPs initiated the questionnaire (see participant flow chart, Figure 1 in Supplemental Material 1). Of these, 3 participants were excluded because they did not meet all the inclusion criteria, and 10 PCPs did not complete the survey. Thirty-nine participants were analysed, resulting in a response rate of 63%. Participants were almost evenly distributed between the two services (service A: *n* = 20; service B: *n* = 19, see Table 2).

**Table 2.** Sample characteristics.

Characteristic	Total (*n* = 39)			Nursing (*n* = 28)		Physicians (*n* = 6)		Other health prof. (*n* = 5)		Service A (*n* = 20)		Service B (*n* = 19)	
		*n*	%	*n*	%	*n*	%	*n*	%	*n*	%	*n*	%
Service	A	20	51.3	17	60.7	2	33.3	1	20				
	B	19	48.7	11	39.3	4	66.7	4	80				
Profession	Registered nurse	27	69.2	27	96.4					16	80	11	57.9
	Physician	6	15.4			6	100			2	10	4	21.1
	Chaplain	3	7.7					3	60	1	5	2	10.5
	Healthcare assistant	1	2.6	1	3.6					1	5	0	0
	Psychologist	1	2.6					1	20	0	0	1	5.3
	Social worker	1	2.6					1	20	0	0	1	5.3
Gender	Female	36	92.3	27	96.4	4	66.7	5	100	20	100	16	84.2
	Male	3	7.7	1	3.6	2	33.3	0	0	0	0	3	15.8
Educational qualification^b^	AFDHE	23	59.0	23	82.1	0	0	0	0	14	70	9	47.4
	Dr	6	15.4	0	0	6	100	0	0	2	10	4	21.1
	MSc or MA	5	12.8	1	3.6	0	0	4	80	1	5	4	21.1
	BSc or BA	4	10.3	3	10.7	0	0	0	0	2	10	2	10.5
	FDVET	1	2.5	1	3.6	0	0	1	20	1	5	0	0
Further Education^a^	CE in PC	15	38.5	7	25	6	100	2	40	5	25	10	52.6
		Mean	Range	Mean	Range	Mean	Range	Mean	Range	Mean	Range	Mean	Range
Degree of employment (in percent)		77.4	25–100	77.3	25–100	83.3	60–100	71	40–95	83	50–100	71.6	25–100
Total work experience (in years)		16.7	2–42	15.6	2–42	16.8	11–26	22.4	5.5–32	15.1	2–42	18.3	5.5–33
Work experience in palliative care (in years) (missing values *n* = 1)		5.7	0.5–35	4.6	0.5–13	7.5	4–12	9.2	3–25	4.6	0.5–13	6.8	0.5–25

a CE in PC: Continuing education programme in palliative care (CAS, MAS).

b AFDHE: Advanced Federal Diploma of Higher Education (equivalent to registered nurse); BSc or BA: Bachelor of Science or Arts; Dr: Doctoral degree; FDVET: Federal Diploma of Vocational Education and Training; MSc or MA: Master of Science or Arts.

### Sample characteristics

The participants, who were predominantly female (92%), came from six different professions, with the majority (72%) being nurses (see Table 2). The degree of employment ranged from 25% to 100% and the work experience in palliative care ranged from 0.5 to 35 years. More than a third of the participants (39%) had a Bachelor of Science or Arts degree or a higher qualification, and 39% had completed continuing education in palliative care.

### PCPs’ skills, attitude towards EBP, and perception of implementation

The PCPs’ self-assessed *skills* in family care (FNPS score), palliative care (EPCS score), and individualised care (ICS-Nurse score) were in the upper quartile of the respective scale range (⩾75%) when considering the median across all participants (see Table 3). For EPCS and ICS-Nurse, the minimum score was over 50%. Self-reported *attitudes* and beliefs towards EBP (EBP-B questionnaire) reached a median of 64%. The highest scoring EBP-B item was “I am convinced that evidence-based guidelines/directives can improve clinical care for patients and their families” (38% “fully agree,” 46% “rather agree”), while the lowest scoring item was “I am convinced that I have the best resources available to implement EBP” (3% “strongly disagree,” 28% “rather disagree”). *Perceived* acceptability (AIM) and appropriateness (IAM) of the BCG each achieved a median score of 75% while feasibility (FIM) reached a median score of 68%.

**Table 3.** Descriptive results of the self-assessment questionnaire.

Domain	Questionnaire	Range per questionnaire	Total sample (*n* = 39)				Nursing (*n* = 28)			Physicians (*n* = 6)			Other health prof. (*n* = 5)			Service A (*n* = 20)			Service B (*n* = 19)			Nurses with CE in PC^a^ (*n* = 7)				Nurses without CE in PC^a^ (*n* = 21)	
			Median	Min	Max	Median percentage^b^ of range (%)	Median	Min	Max	Median	Min	Max	Median	Min	Max	Median	Min	Max	Median	Min	Max	Median	Min	Max	Median	Min	Max
Skills	FNPS^c^	1–5	2	1.2	3.8	75	2.1	1.2	3.8	2.1	1.6	2.3	1.8	1.5	3	2.3	1.4	3.8	1.8	1.2	2.6	2	1.2	3.8	2.1	1.4	3.1
	EPCS	1–5	4.3	3.3	5	82.5	4.3	3.3	5	4.5	4.1	4.9	3.8	3.3	4.5	4.1	3.3	5	4.4	3.3	4.9	4.5	3.5	4.9	4.1	3.3	5
	ICS-Nurse	17–85	74	62	85	87.1	74.5	62	85	75.5	66	83	73	63	77	74.5	62	85	74	63	83	72	63	83	76	62	85
Attitude	EBP-B	16–80	57	41	71	64	56.5	41	71	64	54	67	56	47	67	58	42	64	57	41	67	59	50	71	56	41	67
Perceived implementation	AIM	4–20	16	9	20	75	16	9	20	16.5	14	20	16	14	18	15.5	9	17	16	14	20	16	9	20	16	11	20
	IAM	4–20	16	12	20	75	16	12	20	16	12	18	16	12	17	16	12	17	16	12	20	16	12	17	16	12	20
	FIM	4–20	15	9	20	68	15.5	9	19	16	15	17	13	13	20	15	9	17	16	12	20	14	9	16	16	12	19

AIM: Acceptability of Intervention Measure; EPCS: End-of-life Professional Caregiver Scale; EBP-B: Evidence-Based Practice Belief Scale; FIM: Feasibility of Intervention Measure; FNPS: Family Nursing Practice Scale; IAM: Intervention Appropriateness Measure; ICS-Nurse: Individualised Care Scale for Nurses; PC: Palliative Care.

a Continuing education programme in palliative care (CAS, MAS).

b Median score as a percentage of the scale range: (median score − scale minimum)/(scale maximum − scale minimum).

c Reverse interpretation: lower score equals higher skills, confidence, and knowledge.

### Relationships between PCPs’ skills, attitude, and perception of implementation

The PCPs’ self-assessed palliative care skills (EPCS) were positively associated with their attitudes towards EBP (EBP-B questionnaire; *p* = 0.005, Spearman’s correlation) and the perceived acceptability (AIM, *p* = 0.038), appropriateness (IAM, *p* = 0.029), and feasibility (FIM, *p* = 0.019) of the BCG (Table 4), each to a medium to strong degree given Cohen’s thresholds.^32^ A positive correlation was also observed between acceptability (AIM) and appropriateness (IAM; *p* = 0.002) and between appropriateness (IAM) and feasibility (FIM; *p* = 0.0026).

**Table 4.** Spearman’s correlation for PCPs’ skills, attitude towards EBP, and perception of implementation.

*n* = 39	EBP-B	FNPS^a^	EPCS	ICS-Nurse	AIM	IAM
FNPS^a^	−0.278					
EPCS	0.442*	−0.615**				
ICS-Nurse	0.254	−0.483**	0.644**			
AIM	0.452**	−0.157	0.081	0.048		
IAM	0.349*	−0.249	0.173	0.145	0.482**	
FIM	0.373*	−0.203	0.248	0.235	0.159	0.356*

a Reverse interpretation: lower score equals higher skills, confidence, and knowledge.

AIM: Acceptability of Intervention Measure; EBP-B: Evidence-Based Practice Belief Scale; EPCS: End-of-life Professional Caregiver Scale; FIM: Feasibility of Intervention Measure; FNPS: Family Nursing Practice Scale; IAM: Intervention Appropriateness Measure; ICS-Nurse: Individualised Care Scale for Nurses; PCP: Palliative Care Professional.

* *p* < 0.05. **p < 0.01.

### Differences between professional groups and between services

No evidence of statistically significant differences between the three professional groups was found for any of the seven instruments (see Table 5). A significant difference was found between the two palliative care services in the central tendency of family nursing practice skills (FNPS; *p* = 0.009, Mann-Whitney *U* test), acceptability of the BCG (AIM; *p* = 0.001, Mann-Whitney *U* test), and appropriateness of the BCG (IAM; *p* = 0.022, Mann-Whitney *U* test), with service B scoring higher at the median (lower for the reverse-coded FNPS measure). Nurses without continuing education in palliative care rated feasibility (FIM) higher at the median (*p* = 0.040, Mann-Whitney *U* test).

**Table 5.** Differences between professional groups, services, and levels of continuing education.

Measure	*p* Value (overall null)	*p* Value (D-B) for nurses vs physicians	*p* Value (D-B) for physicians vs others	*p* Value (D-B) for nurses vs others
Differences between professional groups: Kruskal-Wallis chi-squared test (*n* = 39)				
FNPS	0.703	1.000	1.000	0.642
EPCS	0.164	0.260	0.090	0.417
ICS-Nurse	0.621	1.00	0.557	0.556
EBP-B	0.107	0.053	0.232	1.000
AIM	0.779	0.852	0.745	1.000
IAM	0.862	1.000	0.892	0.982
FIM	0.282	0.486	0.167	0.426
Differences between palliative care services: Mann-Whitney *U* test (*n* = 39)				
FNPS	0.009**			
EPCS	0.210			
ICS-Nurse	0.704			
EBP-B	0.278			
AIM	0.001**			
IAM	0.022*			
FIM	0.697			
Differences between nurses with/without continuing education in palliative care: Mann-Whitney *U* test (*n* = 28)				
FNPS	0.915			
EPCS	0.338			
ICS-Nurse	0.352			
EBP-B	0.457			
AIM	0.244			
IAM	0.287			
FIM	0.040*			

AIM: Acceptability of Intervention Measure; D-B: Dunn-Bonferroni pairwise comparison; EBP-B: Evidence-Based Practice Belief Scale; EPCS: End-of-life Professional Caregiver Scale; FIM: Feasibility of Intervention Measure; FNPS: Family Nursing Practice Scale; IAM: Intervention Appropriateness Measure; ICS-Nurse: Individualised Care Scale for Nurses.

* *p* < 0.05. **p < 0.01.

## Discussion

Using a sample from two Swiss hospitals, this study examined the self-assessed skills in family and bereavement care of PCPs in specialised palliative care, their general attitudes towards EBP, and their perceptions of the implementability (acceptability, appropriateness, and feasibility) of newly developed evidence-based BCG. Associations between the PCPs’ attitudes towards EBP and their perception of the implementability of BCG were also examined, showing that more open attitudes towards EBP were associated with higher perceptions of the implementability of evidence-based BCG. No differences were found between professional groups, but some differences were found between the palliative care services and between nurses with different levels of education.

Our results show that PCPs in specialised palliative care largely rate their skills in interacting with and supporting patients and their families (FNPS) and in providing individualised palliative care (EPCS, ICS-Nurse) as high, with the majority in the upper quarter of the respective rating scale. This finding is consistent with other findings of high self-assessed palliative care skills.^22,23,33^ It indicates a sense of confidence, suggesting that skills do not appear to hinder the implementation of bereavement care.^33^ Our findings stand in contrast with studies that identified insufficient skills as a barrier to the implementation of bereavement care.^12,14^ These studies asked about barriers to bereavement care, and a lack of skills was provided as an answer option that was chosen by some of the PCPs surveyed. This discrepancy with our study, which instead asked PCPs to rate their skills, may be because, despite the high self-assessed skill levels of the participants, they still perceived a need for further improvement, particularly more specific training in bereavement care.^12^ Further research is needed to investigate these differences.

The PCPs showed an open general attitude towards EBP that was similar to^34^ or higher^35^ than in other studies. A majority were convinced of its clinical potential, but some did not see sufficient resources available. Their attitudes towards EBP were positively associated with how acceptable, appropriate, and even feasible they perceived the new BCG with a specific pathway to be prior to its implementation. Favourable attitudes towards EBP were also positively associated with self-assessed skills in palliative care (EPCS). This reflects other findings of a positive association between beliefs towards and implementation of EBP in daily practice.^35–37^

Most PCPs rated the implementation of the BCG as highly acceptable (AIM) and appropriate (IAM) before it started, which is known to lead to better implementation,^25,38^ but to a slightly lesser extent as feasible (FIM). This is consistent with another study that used these instruments to evaluate the implementation of a family support intervention.^39^ The perceived acceptability of the BCG and its appropriateness to the specific setting of specialised palliative care correlated to a medium to strong degree, while feasibility correlated to a lesser degree with appropriateness (and not at all with acceptability), which is consistent with the literature.^25^ This is because the assessment of acceptability is specific to the BCG, whereas the assessments of appropriateness and feasibility are also specific to the setting and institution. Even an intervention that is generally perceived as acceptable by the PCPs who are to implement it may be perceived as inappropriate (e.g. not within the mission or competencies of the institution/department) or unfeasible (e.g. insufficient resources) and therefore fail to be implemented. Therefore, the specificity of appropriateness and feasibility for the given setting and institution is crucial for the implementation process in practice as a first step.^40^

These results highlight the importance of an open attitude towards EBP in enabling the successful implementation of EPBs in bereavement care. The literature provides evidence that education and training in applying EBP can improve the perception of and attitude towards EBP.^34,35^ Therefore, educating and training PCP in applying EBP should be used as a basis for successful implementation, underlining the fact that the new BCG is evidence-based. The positive association with the HPCs’ skills in palliative care (EPCS) may be explained by the fact that individuals with a more open attitude towards EBP, such as palliative care,^37^ were more likely to implement it, thereby improving their skills and ranking themselves higher as a result. Improving one’s skills and abilities through implementing new EBP may be a motivating factor.

Our study revealed slight differences between the palliative care services in terms of skills in family care (FNPS) and the acceptability and appropriateness of the BCG, which may indicate the presence of distinct structural enablers^12,14,15^ and emphasise the importance of adapting and tailoring the implementation to the different settings.^40^ To assess these differences between organisational contexts and their implications for the implementation of evidence-based bereavement care, further studies involving the respective structures are needed.

The higher assessment of the feasibility of the BCG among nurses without further training in palliative care is somewhat surprising. Similar observations were made in a Swiss study in which education and training did not promote the feasibility of a dementia care pathway.^41^ Further investigation is required to gain a more profound understanding of these observations.

We did not find a significant difference between the professional groups for any of the outcomes. As is usual with null hypothesis testing of this type, the reverse conclusion that non-rejection of the null hypothesis (*p* > 0.05) proves the absence of a difference/correlation is not permissible. Although there is some evidence of differences in self-assessed end-of-life specific skills in family care by cancer care professionals,^33^ further studies are needed to examine the presence/absence of such differences between professional groups to consolidate and apply them.

### Limitations and strengths

The role of the individual PCP in the BCG was not included in the survey. A strength of this study is the use of validated questionnaires that have been used in other bereavement care studies. The uneven distribution of professions among the survey participants was a challenge for the analysis, but it roughly corresponds to that of other specialised palliative care services in Switzerland and is therefore to be expected.^42^ The sample size was small. However, it should be noted that although this reduces the power to detect correlations/differences (1 minus the probability of a type 2 error), it does not cast doubt on the significant results observed with *p* < 0.05 (type 1 error). When making generalisations from these results, it should be borne in mind that only the palliative care services from two hospitals in the German-speaking part of Switzerland participated, and that the results may therefore only be applicable to similar institutions from similar healthcare systems and similar cultural backgrounds. The current study surveyed PCPs before implementation. Further studies are planned as part of the overall BEST for Family project to investigate how perceptions changed during and after implementation.

## Conclusion

PCPs in the participating specialised palliative care services rated their skills in working with patients and their families overall as high and saw evidence-based BCG as acceptable, appropriate, and mostly feasible, which facilitated the implementation of evidence-based bereavement support. This perception was more pronounced among PCPs who are more open to EBP in general. The implementation of evidence-based bereavement was supported by a general appreciation of the importance of EBP. Perceived implementability and PCPs’ skills were also dependent on the organisational context, which may influence readiness to change. Therefore, hospitals should invest in promoting an EBP culture among healthcare professionals.

## Supplemental Material

sj-docx-1-pcr-10.1177_26323524251369119 – Supplemental material for Palliative care professionals’ perceptions of their skills and the implementability of evidence-based bereavement care guidance: A cross-sectional survey study Supplemental material, sj-docx-1-pcr-10.1177_26323524251369119 for Palliative care professionals’ perceptions of their skills and the implementability of evidence-based bereavement care guidance: A cross-sectional survey study by Sina Gyarmathy, Marco Riguzzi and Rahel Naef in Palliative Care and Social Practice

sj-docx-2-pcr-10.1177_26323524251369119 – Supplemental material for Palliative care professionals’ perceptions of their skills and the implementability of evidence-based bereavement care guidance: A cross-sectional survey study Supplemental material, sj-docx-2-pcr-10.1177_26323524251369119 for Palliative care professionals’ perceptions of their skills and the implementability of evidence-based bereavement care guidance: A cross-sectional survey study by Sina Gyarmathy, Marco Riguzzi and Rahel Naef in Palliative Care and Social Practice
