# Supplementary material for: Palliative care professionals’ perceptions of their skills and the implementability of evidence-based bereavement care guidance: A cross-sectional survey study
Source: Palliat Care Soc Pract. 2025 Sep 16;19:26323524251369119. doi: 10.1177/26323524251369119 (PMC12444063; doi:10.1177/26323524251369119)
Supplement: sj-docx-1-pcr-10.1177_26323524251369119 – Supplemental material for Palliative care professionals’ perceptions of their skills and the implementability of evidence-based bereavement care guidance: A cross-sectional survey study [file sj-docx-1-pcr-10.1177_26323524251369119.docx]

**Palliative care professionals’ perceptions of their skills and the implementability of evidence-based bereavement care guidance: A cross-sectional survey study**

**Supplementary Material 1**

(Authors redacted for peer review)

**Process of recruitment**

Figure 1: Process of recruitment

**
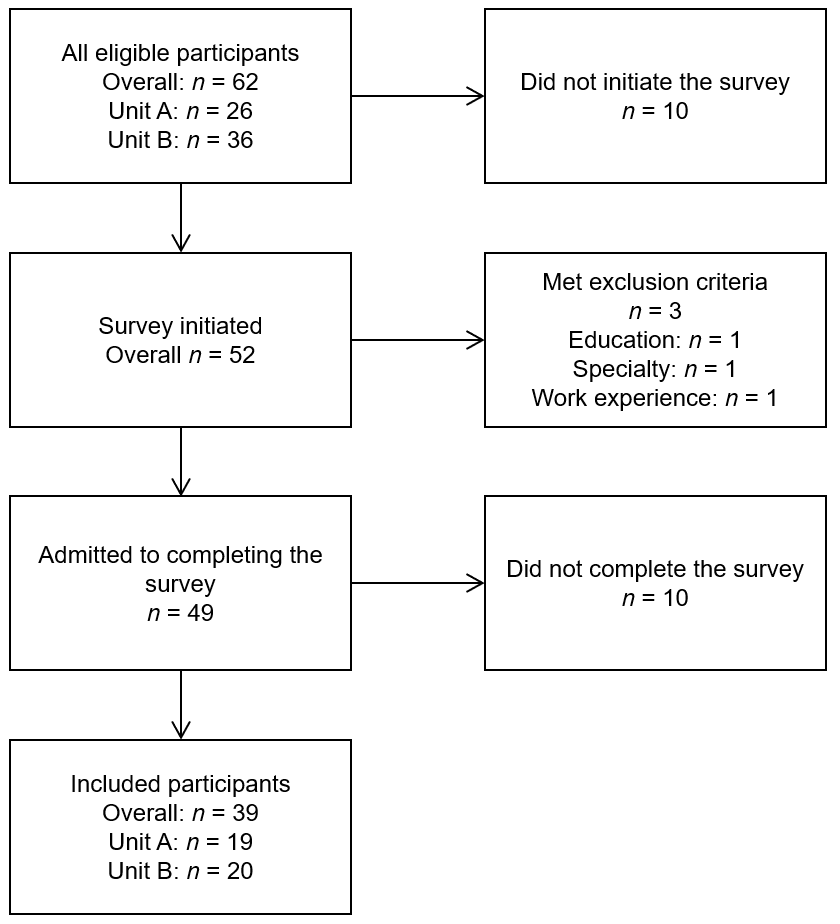
**

**Statistical hypotheses**

Spearman’s pairwise rank correlation coefficient ($\rho$) between palliative care professionals’ (PCPs) self-assessed skill scores (FNPS, EPCS, ICS-Nurse), attitudes towards evidence-based practice (EBP-B questionnaire), and perception of the implementation (AIM, IAM, FIM) tested the null hypothesis of zero correlation versus the two-sided alternative:

$$H_{0}: \rho=0$$

$$H_{1}: \rho\neq0$$

Differences in central tendency of the above-mentioned seven scores between professional groups were tested overall using the Kruskal-Wallis test with the hypotheses

$$H_{0}: \tilde{x}_{1}= \tilde{x}_{2}= \tilde{x}_{3}$$

$H_{1}: \tilde{x}_{i}\neq\tilde{x}_{j}$for at least two groups

and pairwise using the Dunn-Bonferroni post-hoc test:

$$H_{0}: \tilde{x}_{i}= \tilde{x}_{j}$$

$$H_{1}: \tilde{x}_{i}\neq\tilde{x}_{j}$$

Differences in central tendency between palliative care services and between nurses with / without continuing education in palliative care were tested using the Mann-Whitney U-test:

$$H_{0}: \tilde{x}_{1}= \tilde{x}_{2}$$

$$H_{1}: \tilde{x}_{1}\neq\tilde{x}_{2}$$
